# Effects of eri silkworm (*Samia ricini*) pupae inclusion in broiler diets on growth performances, health, carcass characteristics and meat quality

**DOI:** 10.5713/ab.21.0323

**Published:** 2022-01-03

**Authors:** Penpicha Kongsup, Somporn Lertjirakul, Banthari Chotimanothum, Pipatpong Chundang, Attawit Kovitvadhi

**Affiliations:** 1 Animal Health and Biomedical Science Program, Faculty of Veterinary Medicine, Kasetsart University, Bangkok 10900, Thailand; 2 The Queen Sirikit Department of Sericulture, Ministry of Agriculture and Cooperatives, Bangkok 10900, Thailand; 3 Department of Physiology, Faculty of Veterinary Medicine, Kasetsart University, Bangkok 10900, Thailand

**Keywords:** Broiler Chicken, Caecal Digestive Enzyme, Caecal pH, Color, Eri Silkworm

## Abstract

**Objective:**

The objective of this study was to determine the appropriate amount of eri silkworm pupae meal (*Samia ricini*) to add to the broiler diet.

**Methods:**

Two hundred 1-day-old male chicks with initial weight at 50.03±0.56 g/chick were divided into four groups (five replicates per group and ten chicks per replicate): a control group fed a corn-soybean diet and experimental groups supplemented with 5%, 10%, or 15% eri silkworm pupae meal. All experimental diets were isocaloric and isonitrogenous and formulated respecting nutrient requirements. Growth performances were collected during the experimental period and other parameters were collected at the end of experiment when broilers reached thirty-eight days old.

**Results:**

A higher cold carcass weight and skin yellowness in the broilers fed 10% eri silkworm pupae meal compared with the other groups (p<0.05). Therefore, supplementation with 10% eri silkworm pupae meal is suggested for the broiler diet formulation because it did not cause any serious negative consequences on growth performance, health status, carcass characteristics and meat quality. However, the usage of eri silkworm pupae meal at 15% is not recommend because it led to negative outcomes

**Conclusion:**

The addition of eri silkworm pupae at 10% can be used as an alternative protein sources for broiler chickens which provided benefits on cold carcass weight and skin yellowness without adverse effects.

## INTRODUCTION

The broiler chicken is an important livestock with high economic value that generates income for several countries and provides a secure protein source for the ever increasing world population. Broiler production increased around 18.5% from 2015 to 2019. The feed is the major cost of animal production; the protein source represents the highest cost compared with the other raw materials. Generally, fishmeal and soybean meal are used as major protein sources for broiler feed. Fishmeal is an ideal protein raw material because it has a highly digestible and balanced amino acid profile [1–3]. However, fishmeal use in broiler diet formulations has gradually decreased because of several disadvantages regarding nutritional quality fluctuations, pathogen contamination and environmental impacts [1,2]. Therefore, soybean meal has become the major protein source for broilers instead of fishmeal [1–3]. However, soybean meal still has disadvantages because it lacks an essential amino acid (methionine), contains anti-nutritional factors, has low digestibility, leads to environmental deterioration from crop cultivation and can be contaminated with mycotoxins [1,2]. Given these issues, alternative raw protein materials that can be sustainably produced have been studied to replace these current protein sources.

Recently, insects have been of interest to researchers and farmers as sustainable alternative protein sources for broilers and other animal species. A short production cycle, a low investment cost, a small area required for insect production, the possibility that insects can consume by-products, high feed conversion ratio (FCR) and environmental friendliness are the advantages of using insects. Furthermore, using insects as a protein source can decrease the cost of broiler feed [1–3]. Extensive research has shown that several insect species are possible alternative protein sources in broilers: adult Mormon crickets (*Anabrus simplex*), black soldier fly larvae (*Hermetia illucens*), ground crickets (*Gryllus testaceus*), pallid emperor moth larvae (*Cirina forda Westwood*), Chinese grasshopper (*Acrida cinerea*), housefly larvae (*Musca domestica*) and yellow mealworm larvae (*Tenebrio molitor*) [1,2,4–7]. From most of these reports, the growth performance, health status, carcass weight and meat quality were not influenced by using insects in the broiler diet. Moreover, there are antimicrobial peptides in insects that influence the bacterial community and improve broiler health [8,9]. Therefore, insects could serve as alternative protein sources for broilers. There are several insect species that could be produced efficiently and used as protein sources. Therefore, the most suitable insect species, including silkworms, should be selected and studied.

The mulberry silkworm (*Bombyx mori*) has received the vast majority of research attention among silkworm species. Silk is the main valuable product produced by silkworms; their pupae are considered by-products. Mulberry silkworm pupae, muga silkworm pupae (*Antheraea assamensis*), tussore silkworm pupae (*Antheraea mylitta*) and silkworm caterpillars (*Anaphe infracta*) [6,7] are possible alternative protein sources in broiler nutrition. However, some silkworm pupae are consumed by humans and mulberry silkworms can only eat mulberry leaves [6]. Therefore, the cost of mulberry silkworms could be too high for use in broiler feed. Another interesting silkworm species, the eri silkworm (*Samia ricini*), can be produced in an environmentally sustainable manner. This species is resistant to hot climates and its highest production is in Asia and the Pacific region [6,10]. Interestingly, cassava leaves by-products from cassava production are used for eri silkworm feed; these leaves are low cost and support an upcycling economy. The pupal stage contains high quality nutrients and is rich in crude protein (CP), from 49.4% to 54.6% [6,11]. The *in vitro* digestibility of CP based on simulations of the broiler digestive tract is similar to dehulled soybean meal [11]. Moreover, people prefers to consume mulberry silkworm rather than eri silkworms [10]. Given these factors, eri silkworms have the potential for use in broiler feed. However, the appropriate percentage to add in feed and the consequences on broilers are unknown. Therefore, this study evaluated the appropriate concentration of eri silkworm supplementation in broiler feed based on growth performances, health status, carcass characteristics and meat quality.

## MATERIALS AND METHODS

### Care and use of animals

This study was carried out following the standard guidelines approved by the Institutional Animal Care and Use Committee of Kasetsart University, Bangkok, Thailand (ACKU63-VET-010).

### Amino acid profiles determination

The amino acid composition was analysed using high performance liquid chromatography (HPLC) following the standard method of AOAC [12,13]. The ground, freeze dried eri silkworm pupae and dehulled soybean meal were hydrolysed with 6 N HCl for 16 to 18 h at 110°C. HPLC was performed using an Agilent 1290 Infinity II (UHPLC, Böblingen, Germany) with the following features: sample injection, multisample (G7167B); pump, flexible pump (G7104A); column: Agilent Poroshell HPH-C18 with guard column (40°C to maintain temperature for amino acids); detector: diode array detector (DAD; G7116B). Mobile phase A was borate buffer pH 8.2 (filtered through a 0.45 μm regenerated cellulose membrane) and mobile phase B was acetonitrile: methanol:water (45:45:10 v/v/v, all HPLC grade). For injection, 100 mL of mobile phase A was mixed with 0.4 mL of concentrated H3PO4. The flow rate was 200 μL/min. Prior to injection, amino acids were derivatised with *y*-phthalaldehyde and 9-fluorenylmethyl chloroformate for primary amino acids and proline, respectively. Amino acid standard solutions was purchased from Agilent (Santa Clara, CA, USA).

### Insects, animals, husbandry, and diets

Eri silkworm pupae were obtained from the Queen Sirikit Department of Sericulture (Bangkok, Thailand), where they had been boiled, subjected to silk reeling and frozen at −20°C before arriving at the experimental site at the Faculty of Veterinary Medicine, Kasetsart University, Bangkok, Thailand. The eri silkworm pupae were dried at 60°C for 48 h, ground to 1 mm and kept at −20°C for use as an ingredient in experimental diets [11].

Two hundred 1-day-old male broiler chicks (Avian CP; Charoen Pokphand Foods PCL., Bangkok, Thailand) with initial weight at 50.03±0.56 g/chick were randomly assigned into four experimental group with five replicates per group and 10 birds per replicate. Chicks were vaccinated against Newcastle disease and infectious bronchitis by spraying in the hatchery. All chickens were placed in floor cages with rice hull as bedding inside a closed housing system under controlled a lighting programme (18 h light:6 h dark) and able to access food and water *ad libitum*. The temperature of the experimental room was controlled at 34°C for 5 days and gradually decreased by 2°C per week to 26°C, which was maintained until the end of the study at 38 days. The main protein raw material for the control group was soybean meal, whereas the eri silkworm pupae meal was used in the diet formulation at 5% (5%ERY), 10% (10%ERY) or 15% (15%ERY) in the treatment groups. All starter (1 to 21 days old) and finisher (22 to 38 days old) mash diets were isocaloric and isonitrogenous and formulated respecting nutrient requirements. The ingredients and chemical composition of experimental diets was analysed in triplicate [12,13] and the details are presented in Table 1. Of note, the nitrogen-to-protein conversion factor was 4.76 for the eri silkworm pupae meal or 6.25 for the other ingredients [14].

### Growth performances, blood sample collection and slaughtering

Live weight and feed intake (FI) were checked every 2 weeks throughout the experiment to calculate average daily weight gain (ADG), average daily feed intake (ADFI) and the FCR. In addition, the same observer recorded any morbidity and mortality daily. At the end of the experiments (38 days), serum was collected from the brachial wing vein (n = 5 per replicate) to evaluate the levels of aspartate transaminase (AST) and alanine transaminase (ALT). Subsequently, at a commercial slaughterhouse all fattening broilers were weighed to obtain the slaughter weight (SW), shocked by electricity in a water bath and bled by cutting both jugular veins until broilers died [15].

### Carcass characteristics and meat quality analysis

Carcass characteristics and meat quality analysis were adapted from the study by Kovitvadhi et al [15]. The hot carcass weight (HCW) was obtained, and the feet, head, spleen and gastrointestinal tract were weighed after the slaughtering process. The caecal pH was immediately measured and the caecal material was collected and stored at −20°C for further analysis on caecal digestive enzyme activities. The carcass was chilled at 4°C for 24 h, after which time the cold carcass weight (CCW); dressing out percentage; and liver, breast, thigh, wing and meat/bone ratio were determined. The lightness and colour of the skin and pectoralis and gastrocnemius muscle at the same area for each carcass were evaluated with a chroma meter (CR-410; Konica Minolta, Tokyo, Japan). Breast muscle was collected in duplicate to determine meat pH, drip loss after 24 and 48 h and thiobarbituric acid reactive substances after storage at 4°C for 7, 14, and 28 days. The dry matter (DM) and chemical composition of CP, ether extract (EE) and crude ash of freeze-dried pectoralis and gastrocnemius muscles were determined in duplicate. The crude fat was also determined in the broiler liver [12,13].

### Caecal digestive enzyme activities

Caecal digestive enzyme activities were determined based on the study by Kovitvadhi et al [15] with adaptations. The caecal content was homogenised with phosphate-buffered saline (PBS, pH 7.0; 1:3 v/w), centrifuged at 18,000 g for 30 min at 4°C and the supernatant was removed and stored at −80°C as crude enzyme extract (CTX) for further analysis. The caecal digestive enzyme activities were evaluated in duplicate.

For protease activity, 50 μL of 2% casein in 0.2 M NaOH, 200 μL of 0.2 M PBS (pH 6.8) and 125 μL of CTX was mixed and then incubated at 40°C for 15 min. After the incubation, 5% trichloroacetic acid was added to terminate the enzymatic reaction, followed by centrifugation at 5,000 g for 20 min at 40°C. Then, the supernatant was added to 350 μL of 0.5 M NaOH and 250 μL of 5 M Folin-Ciocalter reagent. The absorbance of the mixture was determined at 720 nm. L-tyrosine was used as the reference [15].

To determine amylase activity, 25 μL of 5% soluble starch in distilled water, 62.5 μL of 0.2 M PBS (pH 6.8), 37.5 μL of 20 mM NaOH and 125 μL of CTX were mixed together and then incubated at 40°C for 15 min. After incubation, 250 μL of 1% 3,5-dinitrosalicylic acid (DNS) was added and the mixture was incubated in boiling water for 5 min. Distilled water (1.2 mL) was added after the solution reached room temperature (25°C). The absorbance was measured at 540 nm, with maltose used as the standard [15].

To determine cellulase activity, 75 μL of 1% carboxyl methyl cellulase, 425 μL of 0.2 M PBS (pH 6.8) and 50 μL of CTX were mixed together and incubated at 40°C for 30 min. Then, 300 μL of 1% DNS was added and the solution was boiled in water for 10 min. The absorbance was evaluated at 540 nm, with glucose used as the standard [15].

To determine chitinase activity, 250 μL chitin solution (10 mg of chitin), 250 μL of 0.2 M PBS (pH 6.5) and CTX were mixed and incubated at 40°C for 60 min. After incubation, 300 μL of 1% DNS was added and the solution was incubated in boiling water for 30 min. The absorbance was measured at 560 nm, with glucose used as the standard [16].

### Statistical analysis

A completely randomised design was selected for this study. The control and experimental diets (fixed factors) were compared by one way analysis of variance with Duncan’s multiple range test as the post hoc analysis. The effects of different levels of eri silkworm pupae meal were evaluated by using orthogonal polynomials for linear, quadratic and cubic effects. The normal distribution and homogeneity of variance were confirmed by the Shapiro–Wilk test and Levene’s test, respectively. A difference was considered statistically significant at p<0.05. All statistical analysis in the study was investigated by using R in RStudio ver.1.4.1103 with the Rcmdr package [17].

## RESULTS

### Chemical composition and amino acid profiles in eri silkworm pupae

One hundred grams of eri silkworm pupae on a DM basis (CP = 49.4% of DM using 4.76 as the nitrogen-protein conversion factor) contains aspartic acid, glutamic acid, serine, histidine, glycine, arginine, threonine, alanine, proline, cystine, tyrosine, valine, methionine, lysine, isoleucine, leucine and phenylalanine at 4.86, 3.05, 3.70, 0.78, 3.65, 2.55, 2.14, 2.18, 1.88, not detected, 3.58, 2.48, 1.01, 3.34, 2.03, 3.35, and 2.60 g, respectively. One-hundred grams of soybean meal on a DM basis (CP = 46.5% of DM using 6.25 as the nitrogen-protein conversion factor) contains 4.92, 7.83, 2.11, 1.19, 1.85, 3.21, 1.70, 1.94, 2.20, 0.66, 1.54, 2.11, 0.62, 2.73, 2.04, 3.34, and 2.24 g, respectively

### Growth performances

The productive performance of broilers fed different concentrations of eri silkworm pupae meal was presented in Table 2. There were no differences in the live weight and the ADG among the studied groups (p>0.05). The highest ADFI and FCR at 1 to 14 days were observed in the 15%ERY group (p<0.05). At 29 to 38 days, broilers fed 15%ERY presented a lower ADFI compared with the control and 10%ERY groups (p<0.05). The FCR of 5%ERY and 15%ERY was lower than the control group (p<0.05). In addition, the ADFI and FCR did not differ among the study groups at 15 to 28 days and along the entire experiment (p>0.05). Based on the linear contrast results, supplementation with eri silkworm pupae meal at 10% increased the ADFI at 1 to 14 days and reduced the ADFI at 29 to 38 days (p<0.05). Consequently, the FCR at 1 to 14 and 29 to 38 days was increased and decreased, respectively (p<0.05).

The average final live weight and FCR were not difference between experimental groups (p>0.05). There was no morbidity observed, but one and two broilers in the 5%ERY and 15%ERY group, respectively, died.

### Liver enzymes, caecal pH, caecal digestive enzyme activities and carcass characteristics

Table 3 presents the liver enzymes, caecal pH, caecal digestive enzyme activities and carcass characteristics. The liver enzyme levels (AST and ALT) were not different among the groups (p>0.05). In addition, broilers fed 15%ERY had more liver fat than the other groups (p<0.01). The control group had the lowest caecal pH at the end of the experiment (38 days); all broilers fed eri silkworm pupae meal presented a higher caecal pH than the control group (p<0.01). The caecal amylase activities in the control and 5%ERY groups were higher than the other groups (p<0.001), whereas the control group had the highest caecal protease activity (p<0.001). The caecal cellulase and chitinase activities were not different among the experimental groups (p>0.05).

Interestingly, the group fed 10%ERY had the highest CCW, whereas the group fed 15%ERY had the lowest CCW (p< 0.05). In addition, the HCW and CCW presented a quadratic pattern in which the 10%ERY supplementation showed the highest values; both measures decreased with 15%ERY (p< 0.05). The liver weight of broilers fed 15%ERY was higher than other groups (p<0.001), whereas the control group had a higher gastrointestinal weight compared with the other groups (p<0.001). The 10%ERY group had a lower spleen weight compared with the control and 5%ERY groups (p< 0.05) but 10%ERY group not difference with 15%ERY. The SW and carcass characteristics regarding the HCW, feet, head, dressing out percentage, breast, thighs, wings and meat/bone ratio were not different among the groups (p>0.05).

### Skin color and meat quality

Table 4 shows the skin colour and meat quality of the groups. There was greater redness, yellowness and chroma of the skin in the 10%ERY and 15%ERY groups compared with the control group (p<0.05); the lightness and hue angle did not differ among the groups (p>0.05). The 15%ERY group had lower lightness and greater redness, yellowness and hue angle in the breast (pectoralis muscle) compared with the other groups (p<0.001), whereas the chroma was not different among the groups (p>0.05). The redness and yellowness of the skin and breast meat increased following a linear model as the amount of eri silkworm meal increased (p<0.001). There were no colour differences for the hindlimb muscle (gastrocnemius; p>0.05) except higher lightness in the 5%ERY compared with the control and 15%ERY groups (p<0.05). The 5%ERY group had a higher drip loss percentage after storage for 24 h compared with the control and 15%ERY groups (p<0.05). There were no differences found in meat pH after 24 h, drip loss percentage after storage for 48 h, thiobarbituric acid reactive substances (TBARS) and the chemical composition of the pectoralis and gastrocnemius muscles (p>0.05).

## RESULTS AND DISCUSSION

### Growth performances

Growth performance can vary depending on the insect species used to supplement broiler feed [2,18]. There have been no reports of adverse effects on growth performance when replacing a low amount of fishmeal (2% to 10%) in the broiler diet formulation with house fly larvae, silkworm caterpillar meal (*A. infracta*), westwood larvae, yellow mealworm, grasshoppers or field crickets [2,4–7]. However, if too much insect meal is included in the diet, there can be deterioration of growth performance. In addition, the substitution of insect meal cannot deliver the same outcomes on growth performance as fishmeal [2,4–7].

Soybean meal has become a major protein source in broiler feed, replacing fishmeal in several countries. The partial replacement of soybean meal with black soldier fly larvae meal 10% to 20% in broiler feed [4] or 50% to 100% in layer feed [2,7] did not affect productive performance. Furthermore, Mormon cricket meal was able to completely replace soybean meal without adverse effects. Moreover, the usage of house fly larvae [19] or yellow meal worm larvae [5,20] improved the FCR [21] compared with the control group fed using soybean meal as the protein source. Several studies have investigated the addition of silkworm pupae in broiler feed. The usage of mulberry, muga and tussore silkworm pupae to replace fishmeal at 50% or 5% to 10% in feed formulation did not cause adverse effects on productive performance [6]. In addition, supplementation of around 5% of the feed formulation with silkworm pupae improved the FCR and reduced the production cost. In the same way as other insects, substitution at 15% or 20% in the feed formulation promoted negative consequences on productive performance [1,2, 7,21]. The results in this study are consistent with these findings, especially considering the HCW and CCW. Therefore, the appropriate level of addition is an important consideration regarding which insect species to use in feed.

The growth performance of broilers are influenced not only by level of insect meal addition but also the chemical composition and digestibility of the meal [2,4–6]. The diets used in this study were isocaloric and isonitrogenous. However, the calculated CP was lower than the analysed CP because of the different nitrogen-protein conversion factor used. Insects have chitin, which contains non-protein nitrogen molecules; therefore, the amount of CP should be multiplied by a lower nitrogen-protein conversion factor [14]. Hence, a nitrogen-protein conversion factor of 4.76 was used for the eri silkworm pupae meal, whereas a value of 6.25 was used for the other raw materials. In other studies, the analysed CP was measured in complete mixed diets using a nitrogen-protein conversion factor of 6.25. Based on this formulation, the differences we noted regarding productive performance could be due to the different amino acid profiles and digestibility between the soybean meal and eri silkworm pupae meal and not due to a different CP content.

Similarly to mulberry silkworm pupae, the eri silkworm pupae had lower cysteine compared with soybean meal [6]. In a previous study, broilers fed by partly defatted black soldier fly larvae, which has insufficient cysteine, induced a cysteine-sparing effect due to a lack of methionine and led to a deterioration of growth performance and FI [22]. Fortunately, maggot meal, silkworm pupae, mealworms and eri silkworms contain more methionine than soybean meal [19]. Therefore, the severe negative outcomes of cysteine deficiency were not observed in this study. Moreover, ecdysteroid, an insect hormone for metamorphosis that has growth-stimulating properties, has been found in silkworm pupae [6]. This could be another reason why the FCR was improved in the 5%ERY and 10%ERY groups, whereas 15% eri silkworm pupae meal could represent too much supplementation considering the lack of cysteine and/or methionine. Additional investigation is required to confirm this hypothesis.

Crude fiber, ADF and chitin [1,11] in insect meal are negatively correlated with protein digestibility. Therefore, supplementing broiler feed with insects that contain high levels of these chemical components could lead to lower protein digestion and, consequently, poor performance. Based on *in vitro* digestibility on crude protein from broiler crude enzyme extract, Bombay locust (*Patanga succincta*), housefly larvae, lesser wax moth larvae (*Achroia grisella*) and yellow mealworm were similar to fishmeal and higher than dehulled soybean meal, whereas mulberry and eri silkworm were similar to dehulled soybean meal [6]. Moreover, the digestibility of DM and nutrients were not different between broilers fed a diet with mulberry silkworm pupae or soybean meal. This result could indicate that silkworm pupae have the potential to replace soybean meal. In addition, the digestibility can be improved by enzyme addition or pre-fermentation [7].

### Skin color, carcass characteristics and meat quality

There are very few studies available in the literature on the skin colour of broilers fed insect meal. The use of housefly maggot meal at 10% or 20% in broiler feed did not change the breast meat colour [2]. However, the inclusion of yellow mealworms [20] or black soldier fly larvae [23] led to more lightness, redness and yellowness of breast meat. We also found increased yellowness with the inclusion of eri silkworm pupae meal. Generally, an increase in yellowness is due to pigments in the diet. On the one hand, pigments were observed in most of insects and several classes of pigments are involved with insect colouration. Melanins produce shades from black to reddish-brown, and pterins, ommochromes and carotenoids contribute to red, orange, and yellow colours [24]. Therefore, the increased yellowness in meat and skin could be due to the pigments inside eri silkworm pupae. In addition, insect meals contain a high proportion of medium-chain fatty acids, mainly lauric acid, whereas polyunsaturated fatty acids are mainly found in palm oil, which was used in control diet. Lipid peroxidation is a major concern regarding product quality and safety. TBARS was not different among the studied groups, and thus the inclusion of eri silkworm pupae meal did not cause lipid peroxidation. The fatty acid profiles from diets was similar in meat [23]; therefore, the increased yellowness could due to the insect oil deposited in the broiler’s meat and skin. This change could promote better consumer perception.

Carcass traits of broilers were not changed by supple menting feed with housefly larvae or yellow mealworms [2]. By contrast, the final weight of broilers showed a trend for an increase in the 10%ERY group compared with the other groups, which led to a significant difference in the CCW. On the one hand, broilers fed by supplementation with grasshoppers [24], black soldier fly larvae [23], African palm weevil larvae (*Rhynchophorus phoenicis*). In addition, the breast muscle weight was increased by supplementation of oil from black soldier fly larvae [23].

### Liver enzymes, liver fat, caecal pH and caecal digestive enzyme activities

The liver weight was increased following 15%ERY supplementation; this change was likely due to greater liver function or deposited fat. The levels of enzymes that indicate liver damage, AST and ALT, were not increased in the experimental groups; therefore the supplementation of eri silkworm pupae meal did not lead to hepatic damage. In another study, lipid accumulation in the liver of broilers retarded the production of methionine but did not negatively affect the liver [24]. We speculate that the different fatty acid profiles between palm oil and insect oil could be the cause of this outcome. Based on our results, the usage of 15%ERY longer than 38 days might induce hepatic damage. The damages of hepatic function has been implicated to reduce growth performance and threaten the health of birds, causing economic losses to the poultry industry [25]. Therefore, long-term usage in layers and parent stocks should be carefully considered.

The microbial community correlates to the health status in broilers; this factor can be roughly represented by the caecal pH and digestive enzyme activities. The increase in short-chain fatty acids after microbial fermentation reduces the pH [2]. The control group had a lower caecal pH compared with the experimental groups. The elevated pH is caused by more ammonia, leading to pathogen growth and risk for morbidity [2]. However, the caecal pH of the experimental groups was still in the normal range [2] and none of groups showed morbidity. Therefore, the supplementation did not negatively affect the health status. The differences in amylase and protease activities among the groups indicate modifications to the microbial community. Chitinase is excreted from microbes inside the chicken caecum [2,7]. We expected that the chitinase activity would increase as the level of eri silkworm pupae meal in the feed increased. However, the chitinase activity was not different among the studied groups. Hence, the tested levels of eri silkworm pupae meal did not affect the bacteria that produced chitinase. Therefore, the microbial community inside commercial broilers did not support the increase of chitin level from insects. The appropriate level of insect meals supplementation must be the important consideration point to prevent negative outcomes on lower digestibility and increase FCR. More in-depth studies on the microbial community based on genetic techniques could be performed to confirm whether eri silkworm pupae meal benefits or harms the caecal microbial community.

## CONCLUSION

This study aimed to determine the appropriate level of eri silkworm pupae meal to include in broiler feed. Based on the results, the addition of 10% eri silkworm pupae meal had a CP similar to dehulled soybean meal (26%). Compared with the control group, these broilers presented a higher final weight, a lower FCR and a higher CCW. Interestingly, the yellowness of the skin increased in the 10%ERY and 15%ERY groups compared with the control group (p< 0.01). All broilers fed diets containing eri silkworm pupae meal presented a higher caecal pH compared with the control group (p<0.01). There was a trend for reduced caecal amylase and protease activities compared with the control group. The addition of eri silkworm pupae meal in the broiler diets did not promote serious negative consequences on other growth performance parameters, liver enzymes, carcass traits and meat quality. Based on the study results, we suggest adding 10% eri silkworm pupae meal to broiler feed. The usage of 15% eri silkworm pupae meal is not suggested because it caused negative outcomes: lower final weight, higher FCR, lower CCW and higher liver weight. Additional studies on cost effectiveness, the fatty acid profile in meat and the microbial community could be of interest to confirm the usage of eri silkworm pupae as an alternative protein source for broilers.

## Figures and Tables

**Table 1:** Ingredients and chemical composition of studied diets

Items	Starter diet (1 to 21 d)				Finisher diet (22 to 38 d)			
	
	Control	Eri silkworm addition			Control	Eri silkworm addition		
	
		5%	10%	15%		5%	10%	15%
Ingredients (as-fed basis, %)								
Corn	50.8	54.2	57.6	61.1	62.8	66.1	69.8	73.1
Soybean meal 46.5% CP	39.3	32.7	26.0	19.2	28.0	21.4	14.4	7.80
Eri silkworm powder	-	5.00	10.0	15.0	-	5.00	10.0	15.0
Palm oil	6.06	4.18	2.35	0.41	5.73	3.87	1.99	0.09
Monodicalcium phosphate	1.38	1.31	1.25	1.19	0.91	0.88	0.79	0.75
Limestone	1.38	1.40	1.43	1.45	1.27	1.29	1.33	1.35
Salt	0.24	0.24	0.24	0.24	0.19	0.19	0.19	0.19
Vitamin premix^1)^	0.16	0.16	0.16	0.16	0.16	0.16	0.16	0.16
Mineral premix^2)^	0.20	0.20	0.20	0.20	0.20	0.20	0.20	0.20
L-Lysine HCl	-	0.10	0.20	0.32	0.17	0.28	0.41	0.53
DL-Methionine	0.21	0.24	0.25	0.26	0.26	0.27	0.27	0.28
L-Threonine	-	-	0.05	0.20	0.04	0.09	0.19	0.28
Sodium bicarbonate	0.20	0.20	0.20	0.20	0.20	0.20	0.20	0.20
Choline chloride	0.07	0.07	0.07	0.07	0.07	0.07	0.07	0.07
Calculated composition (% DM)								
Crude protein^3)^	22.3	22.3	22.3	22.3	18.2	18.2	18.2	18.2
Metabolizable energy (kcal/kg)	3,100	3,100	3,100	3,100	3,230	3,230	3,230	3,230
Methionine	0.58	0.58	0.58	0.58	0.53	0.53	0.53	0.53
Methionine+cystein	0.94	0.91	0.87	0.83	0.84	0.80	0.76	0.72
Calcium	0.89	0.89	0.89	0.89	0.75	0.75	0.75	0.75
Available phosphorus	0.45	0.45	0.45	0.45	0.38	0.38	0.38	0.38
Analyzed composition (% DM)								
Crude protein^4)^	22.4	23.1	24.0	24.9	18.3	19.1	19.8	20.7
Ether extract	8.37	7.21	6.05	4.87	8.28	7.15	5.93	4.80
Ash	4.75	4.89	4.76	4.84	4.54	4.44	4.52	4.62
Crude fiber	3.24	3.27	3.30	3.32	3.08	3.11	3.13	3.16

CP, crude protein; DM, dry matter.

1) Vitamin premix (Feed specialties Co., Ltd; Pathumthani, Thailand) were supplied per kilogram of diets at 2,500,000 IU of vitamin A; 1,000,000 IU of vitamin D_3_; 7,000 IU of vitamin E; 700 mg of vitamin K; 400 mg of vitamin B_1_; 800 mg of vitamin B_2_; 400 mg of vitamin B_6_; 4 mg of vitamin B_12_; 30 mg of biotin; 3,111 mg of Ca pantothenate acid; 100 mg of folic acid; 15,000 mg of vitamin C; 5,600 mg of vitamin B_3_.

2) Mineral premix (Feed specialties Co., Ltd; Pathumthani, Thailand) were supplied per kilogram of diets at 10,500 mg of Zn, 10,920 mg of Fe; 9,960 mg of Mn; 3,850 mg of Cu; 137 mg of I; 70 mg of Se.

3) Nitrogen-to-protein conversion factor at 4.76 or 6.25 was used for the insect meal and other ingredients in feed formulation, respectively [21].

4) Nitrogen-to-protein conversion factor for analysed composition was used at 6.25 [19,20].

**Table 2:** Productive performances between broilers fed different addition of eri silkworm

Items	Groups				SEM	p-value			
	
	Control	Eri silkworm addition				ANOVA	Linear^1)^	Quadratic^1)^	Cubic^1)^

		5%	10%	15%					
Live weight (g)									
1 day	50.0	50.0	50.0	50.0	0.126	1.00	0.94	0.99	0.97
14 days	484	503	511	486	5.878	0.33	0.80	0.08	0.68
28 days	1,168	1,138	1,164	1,072	18.51	0.23	0.12	0.39	0.29
38 days	2,275	2,323	2,324	2,202	26.20	0.32	0.35	0.12	0.75
Average daily weight gain (g)									
1–14 days	31.0	32.3	32.9	31.0	0.424	0.28	0.92	0.06	0.64
15–28 days	49.0	44.1	46.7	40.8	1.421	0.21	0.09	0.85	0.21
29–38 days	79.0	84.6	82.8	79.6	1.580	0.58	0.98	0.19	0.68
1–38 days	58.0	58.8	59.8	54.2	0.945	0.17	0.21	0.09	0.40
Average daily feed intake (g)									
1–14 days	38.7^a^	38.4^a^	42.0^a^	50.6^b^	1.706	0.02	0.01	0.13	0.95
15–28 days	84.7	89.8	89.7	91.6	3.141	0.90	0.50	0.82	0.81
29–38 days	147^b^	131^ab^	139^b^	118^a^	3.976	0.04	0.02	0.67	0.09
1–38 days	113	108	111	105	1.394	0.23	0.10	0.90	0.21
Feed conversion ratio									
1–14 days	1.24^a^	1.19^a^	1.28^a^	1.64^b^	0.056	0.01	<0.001	0.03	0.78
15–28 days	1.77	2.04	1.94	2.21	0.069	0.15	0.05	0.95	0.22
29–38 days	1.88^b^	1.56^a^	1.68^ab^	1.48^a^	0.054	0.04	0.02	0.55	0.08
1–38 days	1.96	1.86	1.86	1.95	0.030	0.52	0.94	0.15	0.98

SEM, standard error of the mean; ANOVA, analysis of variance.

1) Orthogonal polynomial contrasts were calculated to determine the effect of dietary eri silkworm levels.

a,b Means with different superscript differ at p<0.05.

**Table 3:** Liver enzymes, liver fat, caecal pH, caecal digestive enzyme activities and carcass characteristics between broilers fed different addition of eri silkworm

Items	Groups				SEM	p-value			
	
	Control	Eri silkworm addition				ANOVA	Linear^1)^	Quadratic^1)^	Cubic^1)^

		5	10	15					
Liver enzymes									
Aspartate transaminase (U/L)	331	379	292	274	17.84	0.16	0.55	0.18	0.84
Alanine transaminase (U/dL)	3.60	4.60	4.60	4.10	0.274	0.53	0.10	0.35	0.20
Liver fat (% dry matter)	18.3^a^	15.6^a^	22.9^a^	40.8^b^	3.294	0.01	0.01	0.05	0.98
Caecal pH at 38 days	6.51^a^	7.12^b^	6.83^b^	6.99^b^	0.058	<0.01	0.02	0.04	0.01
Caecal digestive enzyme activities									
Amylase (U)	0.29^bc^	0.32^c^	0.24^b^	0.16^a^	0.019	<0.001	<0.001	0.02	0.16
Protease (U)	0.59^b^	0.47^a^	0.40^a^	0.36^a^	0.023	<0.001	<0.001	0.45	0.95
Cellulase (U)	0.56	0.63	0.51	0.47	0.035	0.38	0.23	0.28	0.25
Chitinase (U)	0.16	0.14	0.13	0.12	0.007	0.25	0.05	0.83	0.81
Carcass characteristics									
Slaughter weight (g)	2,274	2,327	2,324	2,204	21.77	0.16	0.27	0.05	0.75
Hot carcass weight (g)	1,787	1,820	1,857	1,759	15.79	0.15	0.74	0.04	0.32
Feet (%HCW)	4.93	4.76	4.69	4.75	0.033	0.06	0.04	0.08	0.83
Head (%HCW)	3.75	3.75	3.67	3.63	0.037	0.57	0.18	0.81	0.70
Spleen (%HCW)	0.114^bc^	0.121^c^	0.099^a^	0.103^ab^	0.002	0.01	0.01	0.75	0.01
Gastrointestinal tract (%HCW)	9.74^b^	9.04^a^	8.61^a^	8.67^a^	0.088	<0.001	<0.001	0.02	0.78
Cold carcass weight (g)	1,798^ab^	1,826^ab^	1,871^b^	1,745^a^	16.17	0.04	0.43	0.02	0.19
Dressing out (%)	79.0	79.0	80.4	79.2	0.248	0.11	0.42	0.20	0.06
Liver (%CCW)	2.42^a^	2.49^a^	2.56^a^	2.92^b^	0.041	<0.001	<0.001	0.06	0.39
Breast (%CCW)	21.6	21.9	22.8	23.3	0.271	0.09	0.01	0.81	0.71
Thighs (%CCW)	27.5	26.9	27.0	28.0	0.295	0.55	0.54	0.19	0.93
Wings (%CCW)	9.52	9.78	9.56	10.1	0.095	0.20	0.11	0.54	0.17
Meat/bone ratio	4.39	5.05	4.70	5.01	0.121	0.23	0.18	0.47	0.12

SEM, standard error of the mean; ANOVA, analysis of variance; HCW, hot carcass weight; CCW, cold carcass weight.

1) Orthogonal polynomial contrasts were calculated to determine the effect of dietary eri silkworm levels.

a,b Means with different superscript differ at p<0.05.

**Table 4:** Skin color and meat quality between broilers fed different addition of eri silkworm

Items	Groups				SEM	p-value			
	
	Control	Eri silkworm addition				ANOVA	Linear^1)^	Quadratic^1)^	Cubic^1)^

		5	10	15					
Skin color									
Lightness (L*)	63.6	61.4	63.2	61.3	0.417	0.09	0.16	0.85	0.04
Redness (a*)	13.0^a^	14.4^b^	14.7^b^	14.9^b^	0.229	0.02	<0.001	0.18	0.66
Yellowness (b*)	44.2^a^	49.1^ab^	50.4^b^	53.3^b^	1.004	0.01	<0.001	0.59	0.55
Chroma (C*)	46.2^a^	51.2^ab^	52.6^b^	55.3^b^	0.996	0.01	0.21	0.58	0.57
Hue (H*)	1.28	1.28	1.28	1.30	0.005	0.53	<0.001	0.53	0.56
Meat color (*Pectoralis* muscle)									
Lightness (L*)	58.1^b^	59.0^b^	58.1^b^	54.2^a^	0.365	<0.001	<0.001	<0.001	0.65
Redness (a*)	15.9^a^	15.7^a^	16.4^a^	17.8^b^	0.175	<0.001	<0.001	0.01	0.95
Yellowness (b*)	28.1^a^	27.5^a^	29.7^a^	32.9^b^	0.527	<0.001	<0.001	0.06	0.69
Chroma (C*)	1.05	1.04	1.06	1.07	0.006	0.39	0.14	0.58	0.49
Hue (H*)	32.4^a^	31.8^a^	34.0^a^	37.5^b^	0.510	<0.001	<0.001	0.03	0.71
Meat color (*Gastrocnemius muscle*)									
Lightness (L*)	48.5^a^	51.7^b^	50.8^ab^	48.4^a^	0.479	0.03	0.75	<0.001	0.55
Redness (a*)	19.6	18.9	18.3	20.1	0.270	0.09	0.71	0.02	0.33
Yellowness (b*)	27.1	23.7	25.8	29.5	0.870	0.12	0.24	0.04	0.63
Chroma (C*)	0.90	0.90	0.95	0.97	0.011	0.08	0.02	0.54	0.41
Hue (H*)	34.0	30.5	31.7	35.8	0.816	0.09	0.36	0.02	0.78
Meat pH after 24 hours	5.73	5.85	5.79	5.80	0.030	0.60	0.58	0.36	0.36
Drip loss 24 hours (%)	8.88^a^	10.6^b^	9.87^ab^	9.14^a^	0.214	0.02	0.97	<0.001	0.20
Drip loss 48 hours (%)	17.7	19.7	19.1	18.5	0.384	0.29	0.60	0.09	0.44
Thio-barbituric acid reactive substance after storage at 4°C (μg malondialdehyde/mg) at									
7 d	1.05	0.86	0.84	0.75	0.507	0.72	0.92	0.78	0.78
14 d	1.21	1.06	1.26	1.12	0.273	0.40	0.24	0.09	0.09
28 d	2.44	2.83	2.80	3.45	0.855	0.09	0.59	0.36	0.36
Chemical composition of *Pectoralis* muscle (% fresh matter)									
Moisture	75.6	76.1	75.4	75.9	0.315	0.85	0.68	0.38	0.38
Crude ash	1.98	1.92	2.58	3.07	0.240	0.26	0.77	0.67	0.67
Crude protein	21.4	21.8	23.1	21.9	0.356	0.40	0.30	0.28	0.28
Ether extract	1.86	1.28	1.34	1.39	0.258	0.66	0.55	0.73	0.73
Chemical composition of *Gastrocnemius* muscle (% fresh matter)									
Moisture	76.6	76.3	76.8	76.7	0.237	0.91	0.77	0.49	0.49
Crude ash	1.03	1.23	1.08	1.32	0.060	0.32	0.40	0.18	0.18
Crude protein	20.5	21.6	19.1	19.9	0.431	0.20	0.19	0.07	0.07
Ether extract	2.64	3.12	3.32	3.01	0.220	0.79	0.69	0.91	0.91

SEM, standard error of the mean; ANOVA, analysis of variance

1) Orthogonal polynomial contrasts were calculated to determine the effect of dietary eri silkworm levels.

a,b Means with different superscript differ at p<0.05.
